# Secondary peripheral chondrosarcoma in multiple osteochondromas: a retrospective single-institution case series

**DOI:** 10.1186/s13023-023-03006-8

**Published:** 2024-02-13

**Authors:** Maria Gnoli, Marco Gambarotti, Alberto Righi, Eric Lodewijk Staals, Andrea Evangelista, Morena Tremosini, Evelise Brizola, Marina Mordenti, Manila Boarini, Manuela Locatelli, Elena Pedrini, Luca Sangiorgi

**Affiliations:** 1 Department of Rare Skeletal Disorders, IRCCS Istituto Ortopedico Rizzoli, Bologna, Italy; 2 Department of Pathology, IRCCS Istituto Ortopedico Rizzoli, Bologna, Italy; 3 Department of Orthopaedic Oncology, IRCCS Istituto Ortopedico Rizzoli, Bologna, Italy; 4 General Affair Units, Italy, IRCCS Istituto Ortopedico Rizzoli, Bologna, Italy

**Keywords:** Secondary peripheral chondrosarcoma, Malignant degeneration, Multiple osteochondromas, Rare skeletal disorders, Histological evaluation

## Abstract

**Background:**

Multiple osteochondromas is genetic disorder characterized by the formation of multiple benign cartilage-capped bone tumors, named osteochondromas, during skeletal development. The most feared complication is the secondary peripheral chondrosarcoma, a malignant cartilaginous neoplasm that arises from the chondroid cap of pre-existent osteochondromas. We conducted a retrospective cohort study on patients diagnosed and followed up from 1960 to 2019 to describe the clinical and pathological features of individuals affected by peripheral chondrosarcoma in multiple osteochondromas, to evaluate follow up information and individual outcome and to compare the results with literature. Data, including age, gender, site, histological grade, cartilage cap thickness, surgical treatments, surgical margins, genotype mutational status as well as treatment details were captured from the hospital electronic health records and from Registry of Multiple Osteochondromas. In addition, a complete histological review of all hematoxylin and eosin (H&E)-stained sections has been performed by expert pathologists.

**Results:**

One hundred five of the screened cases were included in the present study. The age at diagnosis of SPC ranges from 13 to 63, with median age at diagnosis of 34 years. The site most frequently affected by malignant degeneration was the pelvis (46 patients, 44%) with higher incidence in male patients (32 males vs.14 females). The second one was lower limbs (including femur, fibula, or tibia), identified in 35 patients. Histological information - available for 103 patients – showed: 59 patients with grade 1; 40 patients had a grade 2 and 4 patients had a grade 3. The most common surgical treatment was the complete resection, followed by debulking, amputation and partial resection. Most of cases did not have recurrence of the disease. Outcome in disease-free survival highlights that a worse course of the disease was associated with histological grade 2 or 3, and partial resection surgery. In most of analyzed cases (94%) a pathogenic variant was identified.

**Conclusions:**

In conclusion, the present study gives an overview of the secondary peripheral chondrosarcomas, confirming that this disease represents an impacting complication for multiple osteochondromas patients and suggests that malignant transformation can occur also in younger patient, in a not irrelevant number of cases.

## Introduction

### Background

Multiple Osteochondromas (MO – MIM# 133,700 and MIM# 133,701) [1, 2] is an autosomal dominant rare bone dysplasia frequently defined as Hereditary Multiple Exostoses with a European-based incidence of at least 1:50.000. The representative clinical hallmark are osteochondromas (OCs), benign cartilage-capped bony tumors, that arise on long bones [3]. OCs grow in size and number during childhood until skeletal maturity [1, 4, 5]. Germline pathogenic variants either on *EXT1* [MIM *608177] or *EXT2* [MIM *608210] have been identified in the vast majority of MO patients [6–8]. Since *EXT1* and *EXT2* encode for Golgi-resident glycosyltransferases in charge of heparan sulfate chain synthesis, pathogenic variants on those genes result in systemic heparan sulfate deficiency, impacting on can affect diverse physiologic processes [3].

Malignant degeneration of an osteochondroma in chondrosarcoma is the most serious complication, which is estimated to occur in 0.5–5% of patients [1]. An increase in size of a lesion after puberty and the presence of pain in adults are signs that raise suspicion of a Secondary Peripheral Chondrosarcoma (SPC). In addition, the most reliable feature to suspect a SPC is the cartilaginous cap thickness (exceeding 1.5–2 cm) [9]. Moreover, the imaging or the histology per se may not be distinguish an osteochondroma from low-grade peripheral chondrosarcoma because the diagnosis needs specialized bone tumor pathologists as well as a multidisciplinary approach [10].

At present, the real overall incidence of this event in MO and the risk by age, sex, genotype, and skeletal site is not well known, because of the variability of these values in medical literature [2, 11, 12], even if the male gender tends to have a higher risk of malignant transformation [13].

The available studies in medical literature often did not provide detailed information about malignant transformation in MO or suffered by some bias; in particular, some studies are based on anonymous web-based survey, with self-reported information by the patient, without follow-up details. Other studies were performed on small series or large series collected in tertiary referral institutions; these populations can be not representative of MO patients, so the inclusion of milder forms is limited, and patients not requiring medical treatment or not diagnosed forms are missing [2, 12, 14–17]. While information is available about survival in chondrosarcoma [18], no specific literature on survival rates for chondrosarcomas in MO series is available [19].

### Rationale

The aim of the present study is to evaluate a series of 105 MO adult and adolescent patients - stratified by gender - who underwent chondrosarcoma diagnosis with regard to the role of some factors (i.e., age, site, histological grade, cartilage cap thickness, types of surgical treatment, status of surgical margins, *EXT1/EXT2* genotype mutational status) in risk of developing this complication, and the relationship of these factors to individual outcome.

## Patients and methods

### Study design and data collection

We conducted a retrospective cohort study in SPC cases diagnosed at the IRCCS Istituto Ortopedico Rizzoli from 1960 to 2019. Clinical and radiological data, treatment details and follow-up information were captured from the hospital (electronic) health records and from the Registry of Multiple Osteochondromas (Registro delle Esostosi Multiple, REM - NCT04133285) [20] and paper-based information were digitalized for the aim of the present study.

The presence of osteochondromas was confirmed clinically and radiologically and the diagnosis was made in a multidisciplinary setting (clinical, radiological, pathological experts as well as surgeons), considering individual case characteristics (i.e. age at diagnosis). This series included patients with adequate available material, that has been entirely revised for all histological features, applying to all samples the criteria published by the World Health Organization Classification of Tumors of Soft Tissue and Bone (WHO 2020). In fact, all H&E-stained sections were re-evaluated by two pathologists. The histological grade was confirmed or re-assessed as follow: the diagnosis of grade 1 peripheral chondrosarcoma was made according to the criteria described in the last WHO (2020). The most important feature was the thickness of the cartilaginous cap > 2 cm; a lobular pattern with nodules in the surrounding soft tissue, separated by the mail mass, and the invasion of the stalk of the osteochondroma were also in favor of malignancy. Mitosis and nuclear pleomorphism in such setting defined grade 2 and 3.The types of surgical treatment (complete resection, partial resection, debulking and amputation) has been decided taking into account the definition of margins of the Musculoskeletal Tumor Society as defined by Enneking et al. [21]: wide (the presence of normal tissue between tumor and margin), marginal (resection along the pseudocapsule or reactive zone around the tumor), intralesional (macroscopic or microscopic tumor at the margin) and radical (an entire anatomical compartment excised).

The anatomical site of the neoplasms was established by radiological evaluation. Maximum linear dimension and cartilaginous caps of tumors were measured either radiologically or by gross examination, when possible.

The present study was approved by the independent review committee “Comitato Etico di Area Vasta Emilia Centro” in November 2020 (929/2020/Oss/IOR) and performed in accordance with the ethical standards in the 1964 Declaration of Helsinki.

### Molecular screening

Molecular screening results, performed between 2004 and 2020, were available for 50 cases. All coding exons and flanking exon-intron junctions of *EXT1* and *EXT2* were evaluated by Sanger sequencing using the ABI PRISM 3500XL Genetic Analyzer (Thermo Fisher Scientific, Waltham, MA, USA) for the presence of point mutations; in case of negative results, both genes were evaluated by MLPA (Multiple Ligation-dependent Probe Amplification) analysis for the presence of exon or multi-exons deletion/amplification as previously described [22].

### Statistical analysis

Patients’ characteristics, stratified by gender, were reported using medians (with interquartile range, IQR) and percentages for continuous and categorical variables, respectively. For each patient, the Disease-Free Survival (DFS) was calculated from the date of diagnosis to local recurrence, metastasis (MTS) onset or death as a result of the disease. DFS were estimated with the Kaplan-Meier method and then compared between groups with the log-rank test. The Cox proportional hazard model was used to estimate the Hazard ratios (HRs). A multivariable Cox model for DFS by including all evaluated patients was estimated using multiple imputation to account for missing data in patient characteristics. The combined estimates were then obtained from 25 imputed data sets. A p-value < 0.05 was considered statistically significant. All analyses were performed with Stata 11.2 (StataCorp®, College Station, TX, USA).

## Results

This study includes 105 MO - for the large majority Caucasian Italian - patients − 74 male and 31 female - affected by SPC - that were evaluated at the IRCCS Istituto Ortopedico Rizzoli. Overall, 888 OCs that underwent to surgical intervention at IRCCS Istituto Ortopedico Rizzoli were screened, among them 215 where chondrosarcomas arising from solitary osteochondromas, while 105 (11.82%) were diagnosed as SPC in MO. A summary of clinical, pathological, and molecular information is described in Table 1.

**Table 1:** Patients characteristics by gender. 13 years follow-up time from histological report

Characteristics		Female	Male	Overall
		(N = 31)	(N = 74)	(N = 105)
Histological report years, n (%)				
< 1981		2 (6%)	17 (23%)	19 (18.1%)
1981–1990		2 (6%)	10 (14%)	12 (11.4%)
1991–2000		7 (23%)	14 (19%)	21 (20.0%)
2001–2009		7 (23%)	13 (18%)	20 (19.0%)
> 2009		11 (35%)	20 (27%)	31 (29.5%)
Not Available		2 (6%)	0 (0%)	2 (1.9%)
SPC^*^ age onset, year	Median (IQR)	37 (30, 50)	33.5 (25, 43)	34 (26.5, 44)
Histological grade, n (%)				
1		22 (71%)	37 (50%)	59 (56.2%)
2		7 (23%)	33 (45%)	40 (38.1%)
3		1 (3%)	3 (4%)	4 (3.8%)
Not Available		1 (3%)	1 (1%)	2 (1.9%)
Axial site, n (%)				
No		12 (39%)	33 (45%)	45 (42.9%)
Yes		19 (61%)	41 (55%)	60 (57.1%)
Cartilage cap thickness, cm	Median (IQR)	3 (2.5, 5.5)	3 (2, 5.75)	3 (2.2, 5.5)
Diameter, cm	Median (IQR)	10.5 (8, 15)	11 (8.5, 14.3)	11 (8.5, 14.6)
Surgical treatment, n (%)				
Amputation		5 (16%)	10 (14%)	15 (14.3%)
Complete resection		14 (45%)	30 (41%)	44 (41.9%)
Debulking		8 (26%)	21 (28%)	29 (27.6%)
Partial resection		1 (3%)	8 (11%)	9 (8.6%)
Not Available		3 (10%)	5 (7%)	8 (7.6%)
Recurrence/Metastasis, n (%)				
No		27 (87%)	56 (76%)	83 (79.0%)
Yes		4 (13%)	18 (24%)	22 (21.0%)
Death cause, n (%)				
Alive		31 (100%)	66 (89%)	97 (92.4%)
Dead for disease		0 (0%)	3 (4%)	3 (2.9%)
Dead for other causes		0 (0%)	5 (7%)	5 (4.8%)
Germinal variant, n (%)				
No		1 (3%)	2 (3%)	3 (2.9%)
Yes		13 (42%)	34 (46%)	47 (44.8%)
Not Available		17 (55%)	38 (51%)	55 (52.4%)
Gene, n (%)				
*EXT1*		10 (77%)	21 (62%)	31 (66%)
*EXT2*		3 (23%)	13 (38%)	16 (34%)
Variant type, n (%)				
Big Rearrangement		0 (0%)	4 (12%)	4 (9%)
Deletion		1 (8%)	1 (3%)	2 (4%)
Duplication		1 (8%)	2 (6%)	3 (6%)
Frameshift		4 (31%)	14 (41%)	18 (38%)
Missense		3 (23%)	1 (3%)	4 (9%)
Nonsense		3 (23%)	10 (29%)	13 (28%)
Splicesite		1 (8%)	2 (6%)	3 (6%)

^*^ *SPC: Secondary peripheral chondrosarcoma*

### Demographical features

The age at diagnosis of SPC ranges from 13 to 63 years, with an overall median age at diagnosis of 34 years (IQR 26.5–44). In male patients, the median age at diagnosis of SPC was 33.5 years, while in female patients was 37 years. Cases from 20 to 40 years old represent the 60% of the cases.

Our series comprises also patients younger than 30 years, including, 7 teenager patients had low grade SPC, except one. Six cases had pelvic region involvement (pelvis or proximal femur), and one case of vertebral SPC who underwent to partial resection and showed a recurrence of the disease about 10 years later.

Among 105 patients, 32 (26 males and 6 females) were under 30 years (30%); in 18 of them the pelvis region or the proximal femur were involved and 16 developed grade 2 chondrosarcomas.

Fifty-four patients were between 30 and 49 years old (56.7%); the most affected site is the pelvis (26 subjects), followed by 11 patients with involvement at femur.

Among the 18 older patients – over 50 years of age – the most frequent SPC site is the pelvis (10 patients).

About 30% of cases were over 40 years old and among them the most (60% of case) had a low-grade lesion.

Only 6 patients were older than 50 years had a grade 2 or 3 lesion. Five out of six had a grade 2 SPC, while one developed grade 3 tumor.

### Clinical, surgical, and histological features

About the site of malignant degeneration, axial skeleton was involved in 57% of cases. Pelvis was affected in the most of cases (46 patients, 44%) with higher incidence in male patients (32 males vs.14 females, 70% and 30%, respectively).

The second most frequent site was lower limbs (femur or fibula or tibia), identified in 35 patients (33%), 23 males (65.7%) and 12 females (34.3%), in particular, proximal femur was involved in 17 cases.

The upper limb (6 cases) and the spine (6 cases mainly involving cervical vertebrae (C3, C4, C5 and C7)) represented about 6% of cases, followed by scapula (5 patients). The rib was the site of malignant transformation in 3 cases (3%). A patient had a malignant transformation of calcaneal region OC and another patient at clavicle (Tables 2 and 3).

**Table 2:** Sites affected by secondary peripheral chondrosarcoma

Site	N.	%
Pelvis	46	43.81%
Lower limb^*^	35	33.33%
Proximal femur	17	14.66%
Upper limb^#^	6	5.71%
Vertebrae	6	5.71%
Scapula	5	4.76%
Rib	3	2.86%
Calcaneus	1	0.95%
Clavicle	1	0.95%
Unspecified Site	2	1.90%

^*^ *Lower limb included: femur, tibia, fibula*

^#^ *Upper limb included: humerus, radius, ulna*

Taking into consideration the tumor grade, histological information was available for 103 patients: fifty-nine patients (57%, 37 males and 22 females) had grade 1 SPC; 40 patients (39%, 33 males and 7 females) had a grade 2 and just 4 patients (3 males and 1 female) had a grade 3.

The mean age of diagnosis in grade 1 SPCs was 37 years, 35 years for grade 2 and 38.5 years for the grade 3 cases.

**Table 3:** Patients’ characteristics by site

Characteristics		Upper Limb (N = 11)	Lower Limb (N = 36)	Pelvis (N = 46)	Trunk (N = 10)	*p*-value^*^
Gender, n (%)						
Female		3 (27%)	9 (25%)	15 (33%)	3 (30%)	0.92
Male		8 (73%)	27 (75%)	31 (67%)	7 (70%)	
SPC^#^ age onset, year	Median (IQR)	31 (22, 37)	30 (24, 39.5)	38 (31, 48)	37.5 (32, 43)	0.073
Histological grade, n (%)						
1		7 (64%)	19 (54%)	26 (57%)	6 (60%)	0.19
2		4 (36%)	14 (40%)	20 (43%)	2 (20%)	
3		0 (0%)	2 (6%)	0 (0%)	2 (20%)	

^*^ *Fisher exact test for categorical variables (gender, histological grade), Kruskal-Wallis test for age.*

^#^ *Secondary peripheral chondrosarcoma.*

In 69 cases out of 103, the cartilaginous cap thickness was captured, with a median width of 3 cm, ranging from 2.2 to 5.5 cm.

Diameter of the SPC lesion ranges from 8.5 to 14.6 cm (median 11 cm) for the 78 cases where this information was available.

Information about surgical treatment was provided for 97 patients, 44 of them (45.36%) had a complete resection of the lesion, 9 of them (9.28%) had a partial resection, 29 (29.90%) debulking and 15 (15.46%) amputation.

Taking into consideration the follow-up, 83 of 105 cases (79%) did not have recurrence of the disease neither metastasis, instead a local recurrence was reported in 22 cases (21%). Eight patients (all males) were known to have distal metastasis, in these cases the affected site was pelvis or proximal femur, and the grade was 2 for most of them (7 cases).

**Fig. 1:**
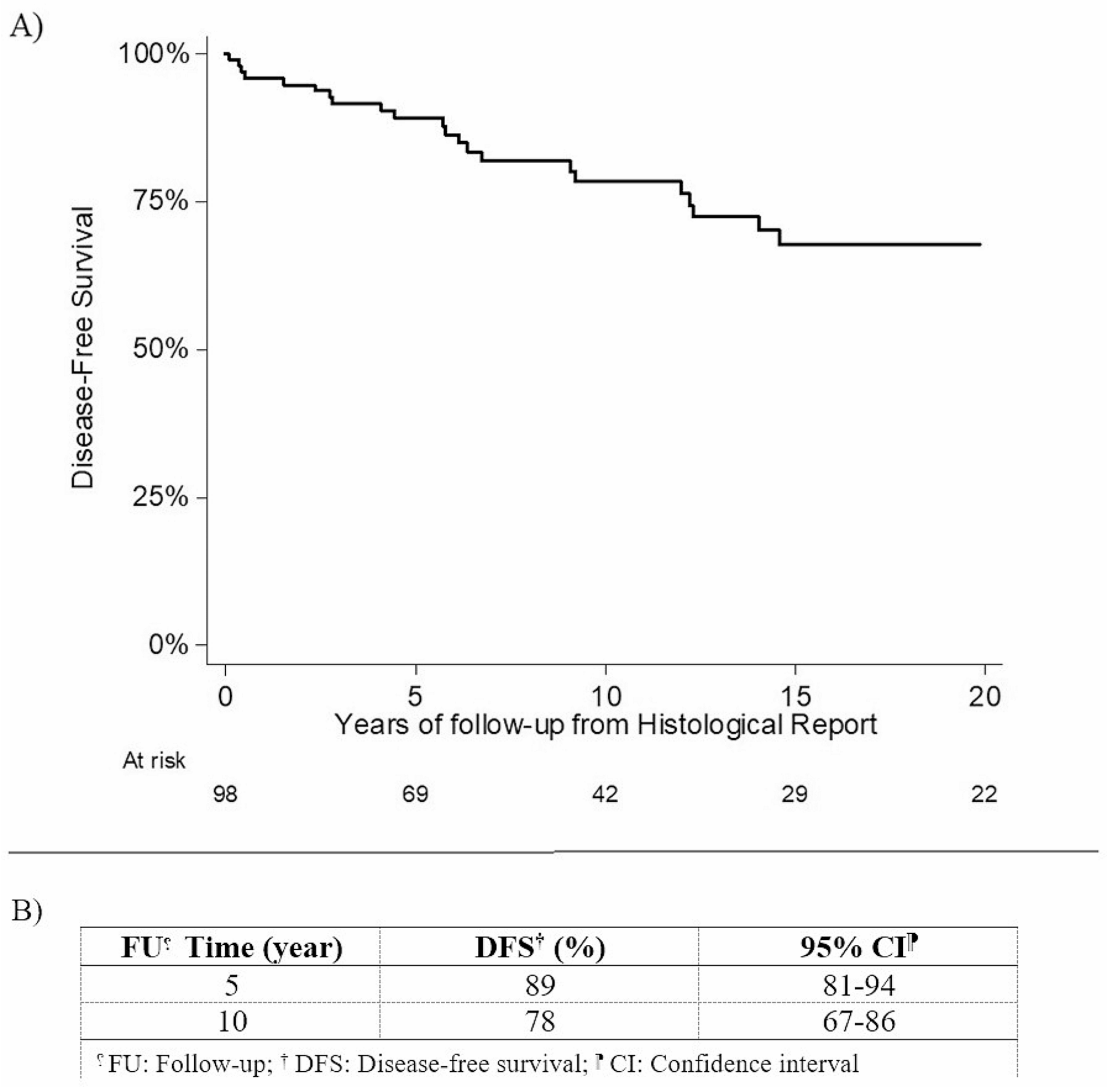
Disease-free survival from histological report. **1A**: Graphical Disease-Free Survival for the entire follow up period. **1B**: Detailed data at 5 and 10 years of follow up from histological reports

As outlined by the Fig. 1A-B, we observe a statistically significant different outcome in Disease-Free Survival in cases treated with a more radical surgery respect to partial resection.

**Fig. 2:**
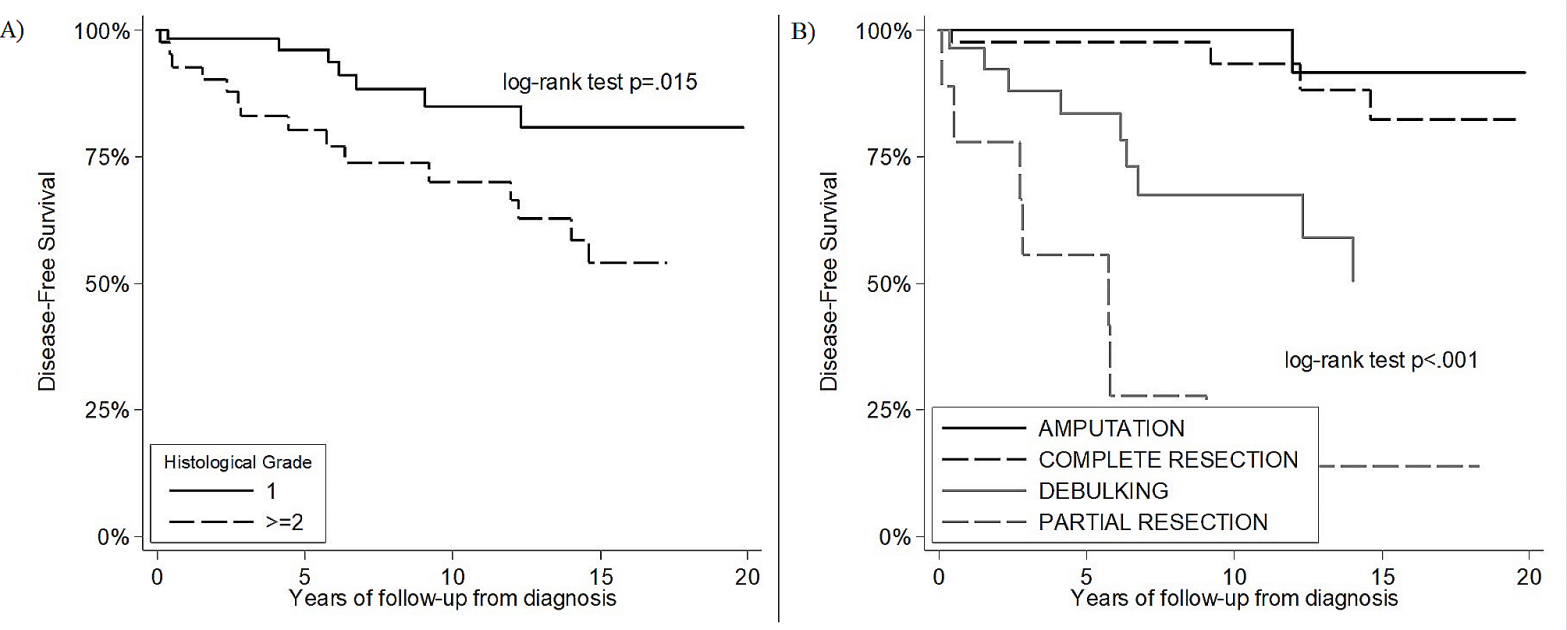
Disease-free survival from histological report considering factors associated to worse disease course. **2A**: Histological grade. **2B**: Type of surgical treatment

At the latest follow-up (December 2020), 97 patients were alive (66 males and all 31 females), while 8 patients died, 3 of them due to the disease. Factors associated to a worse course of the disease were histological grade 2 and 3, and partial resection surgery. Multivariable analysis on Disease-free Survival (DFS) (Recurrence/MTS free Survival) showed that histological grade ≥ 2 (HR 5.22, p 0.003) and partial resection (HR 34.47, p < 0.001) were significantly associated to recurrence of the disease (Fig. 2A-B).

### Molecular evaluation and inheritance

Molecular results of *EXT* genes analysis were available for 50 patients.

In 47 cases (34 males and 13 females), a pathogenic variant was identified, in particular 31 patients (66%, 21 males and 10 females) carry a disease-causing variant on *EXT1* gene, while an *EXT2* causative variant was found in 16 patients (34%, 16 males and 3 females). No pathogenic or unknown significance variants were identified on *EXT* genes in 3 patients (6%, 2 males and 1 female). Variant are listed in Table 4.

**Table 4:** List of identified pathogenic variants

Patient ID	Pathogenic variant	Gene	DNA variant	Predicted aminoacid change
1	YES	EXT2	c.124del	p.Met42Cysfs*17
2	YES	EXT1	c.1018C>T	p.Arg340Cys
3	YES	EXT1	c.652_665del	p.Lys218Tyrfs*2
4	YES	EXT2	c.940-1G>A	
5	YES	EXT1	c.1633insTTTCC	p.Met546Serfs*3
6	YES	EXT1	c.808A>T	p.Arg270Trp
7	YES	EXT1	c.1469del	p.Leu490Argfs*9
8	YES	EXT2	c.1173+1G>A	
9	YES	EXT1	c.2053C>T	p.Gln685*
10	YES	EXT1	c.19dup	p.Tyr7Leufs*23
11	YES	EXT1	c.1225C>T	p.Gln409*
12	YES	EXT1	exon4-11deletion	
13	YES	EXT2	c.514C>T	p.Gln172*
14	YES	EXT1	c.934del	p.Cys312Valfs*47
15	YES	EXT1	c.1054C>T	p.Gln352*
16	YES	EXT1	c.2075G>A	p.Trp692*
17	YES	EXT1	c.357_358insTA	p.Pro120Tyrfs*17
18	YES	EXT2	c.564_566del	p.Leu188del
19	YES	EXT1	c.1705del	p.Val569Cysfs*52
20	YES	EXT1	c.348_349insCT	p.Tyr117Leufs*20
21	YES	EXT1	c.1162C>T	p.Gln388*
22	YES	EXT1	c.1162C>T	p.Gln388*
23	YES	EXT1	c.1057-3C>G	
24	YES	EXT1	c.786C>G	p.Tyr262*
25	YES	EXT1	c.45_46del	p.Cys16Serfs*13
26	YES	EXT1	c.2038G>T	p.Glu680*
27	NO			
28	YES	EXT1	c.1019G>A	p.Arg340His
29	YES	EXT1	c.218del	p.Asn73Thrfs*63
30	YES	EXT1	c.742_743del	p.Arg248Glyfs*40
31	NO			
32	YES	EXT1	c.364C>T	p.Gln122*
33	YES	EXT1	c.364C>T	p.Gln122*
34	YES	EXT1	c.1469del	p.Leu490Argfs*9
35	YES	EXT2	c.67C>T	p.Arg23*
36	YES	EXT1	c.1342dup	p.His448Profs*24
37	NO			
38	YES	EXT1	c.1948delGCCAATTG	Ala650*
39	YES	EXT2	c.87T>G	p.Met87Arg
40	YES	EXT2	c.544C>T	p.Arg182*
41	YES	EXT2	exon4deletion	
42	YES	EXT2	c.314delA	p.Lys105Argfs*7
43	YES	EXT2	c.772C>T	p.Gln258*
44	YES	EXT1	c.906dup	p.Asp303Argfs*11
45	YES	EXT2	exon5-7deletion	
46	YES	EXT2	c.834delG	p.Glu278Aspfs*4
47	YES	EXT2	c.317_318ins	p.Tyr107Valfs*11
48	YES	EXT2	exon1-7deletion	
49	YES	EXT2	c.905del	p.Lys302Serfs*30
50	YES	EXT1	c.1468dup	Leu490Trpfs*9

Information about the family history was available for 53 out of 105 cases of chondrosarcoma. Thirty-seven patients (28 males and 9 females) inherited the disease from a parent; none of the females’ patients had recurrence of disease and 3 out of 28 males were found to have a recurrence of the disease after surgery.

In the subset of patients with molecular analysis, no strong genotype-phenotype correlation can be made, in particular on SPC, mainly due to the limited number of cases.

## Discussion

Malignant transformation is known to be the most serious complication of MO disease. A higher risk of SPC in patients with multiple osteochondromas is known since 1983 [23]. Nevertheless, the real incidence, the age of onset, and the specific risk factors for chondrosarcoma development in MO are at present not well assessed. In fact, few studies of large series or systematically focused on SPC are available in medical literature [19].

In this paper we describe clinical and histological findings and genetic background of a large series of SPC in MO patients.

This study has some limitations. At first, being a reference center of expertise for MO in Italy and a surgery institution, our series can have a selection bias for patients who underwent surgery, with either a higher suspect of malignant degeneration, or a more severe phenotype with a more frequent indication for surgery of an OC. This can negatively impact on number of milder cases (characterized by less health complaint and no need of surgical intervention), leading to a series not completely representative of whole MO population. On the other hand, the evaluated series is very large, including 105 SPC in MO.

The overall chondrosarcoma prevalence we calculated did not encompass these cases in general MO populations, consequently, can be overestimated. At the other hand, because the diagnosis was performed in the same Institution, with expertise in the disease, all the cases are evaluated with homogeneous criteria.

In addition, almost 60 cases underwent surgical treatment before 1990’s, so clinical, imaging and follow-data were collected with different criteria, nevertheless all available pathological samples have been revised by expert pathologists.

In this study we observe a median age at SPC diagnosis of 34 years in line with the reported in medical literature: the review by Fei and colleagues described that 80% of the chondrosarcoma cases occurs before the age 40 (between ages 20 and 40) [19]. This highlighted that onset of this complication in MO is anticipated than the primary chondrosarcomas (ages 30–60) [24]. Previously mean age of degeneration onset in MO was estimated at 31 years [25], moreover, some following reports individuated different age of onset, describing a mean age of about 29 years (± 9.3 years), [14] or 35 years [26].

SPC onset in MO is thought to occur seldom before the 10th or after the 50th year, but occasionally chondrosarcoma was reported in childhood [27].

In our series the 60% of patient were aged from 20 to 40 years, slightly less than reported in some studies in literature [19]. In addition, 32 cases were younger than 30 years and almost the 7% of the cases were younger than 20 years. Even if the median age is in line with medical literature, the present series highlighted the presence of younger patient affected by secondary peripheral chondrosarcoma, particularly because some of these cases are grade 2 SPC. This finding highlights the need of a personalization of follow-up management for young borderline cases.

The higher prevalence of males in this series is in line with literature [13]. This is according to a more severe disease in male patients, moreover in Pedrini and colleagues’ study [17] an almost equal sex ratio was reported in contrast to a recent review [19] and a significant trend toward a more severe phenotype in males was described [1].

According to literature, the most common sites were pelvis and proximal femur, even if in this series we observed scapula involvement in only 5% versus 12% reported in medical literature and spine was the site of malignant transformation in 6% of cases versus 8.6% in the literature review [19]. Pelvis, lower limb, and spine account up to 80% of the cases in the present study, in line with literature data [19]. The higher risk of malignant transformation at the pelvis can be related to its anatomical complexity and adjacency to neurovascular and visceral bundles as per literature [28] In addition, the first suspect for malignant transformation is the increase of size of an OC; if this lesion is in a deep site, only imaging studies can show this variation, so pelvis, more than any other bone district, can allow the tumor can grow before detection.

In literature, cap thickness is a factor capable to differentiate benign osteochondromas versus chondrosarcomas [9]. In our study, the measurement of cap thickness and the disease severity did not result as statistically related, considering the availability of data was for 69 cases.

Instead, in the present study, the higher histological grade (≥ 2) and the partial resection were confirmed to have a prognostic value: both related to worse course of the disease with lower DFS (recurrence/MTS free survival). This data is in line with previous studies, focused on chondrosarcoma, that revealed histologic grade and surgery as two important disease prognostic factors [29, 30].

The disease progression in terms of local recurrence or metastasis was described in about the 20% of the cases, according to a reported survival rate in secondary chondrosarcoma of 75% in a large Mayo Clinic study, even if not focused on MO [11].

Some reports described a more severe phenotype and a higher risk of malignant transformation in *EXT1* variant carriers [2, 31–34], but this finding has not been confirmed in other large series [17]. In the present study, *EXT1/EXT2* genotype was known in less than half of the patients, so it is not possible to infer if the carriers of pathological variants in *EXT1* had actually an increased risk to malignant transformation or other genotype-phenotype correlation. In 6 patients with recurrence of the disease, molecular analysis was available and about equal distribution of variants on *EXT1* and *EXT2* genes was observed.

## Conclusion

These data confirm that SPC in MO patients is an impacting complication, particularly in male, and suggest that malignant transformation can occur also in younger patient, in a not irrelevant number of cases. In addition, our data highlight that OCs more frequently undergo to malignant transformation in some sites, like pelvis and complete resection is the most common surgical treatment. Moreover, our results show that a worse course of the disease is associated with higher histological grade. In conclusion, that knowledge is crucial to better define the optimal screening type for MO patients, nevertheless further studies will be recommended.

## Data Availability

The data supporting the conclusions of this manuscript will be made available by the corresponding authors on a reasonable request. The data are not publicly available due to national privacy regulations.

## Abbreviations

DFS: Disease-Free Survival
HR: Hazard Ratio
H&E: Hematoxylin and Eosin
IQR: Interquartile Range
MLPA: Multiple Ligation-dependent Probe Amplification
MO: Multiple Osteochondromas
MTS: Metastasis
OC: Osteochondromas
REM: Registry of Multiple Osteochondromas
SPC: Secondary Peripheral Chondrosarcoma
WHO: World Health Organization
